# Recent Trends in Nano-Fertilizers for Sustainable Agriculture under Climate Change for Global Food Security

**DOI:** 10.3390/nano12010173

**Published:** 2022-01-05

**Authors:** Krishan K. Verma, Xiu-Peng Song, Abhishek Joshi, Dan-Dan Tian, Vishnu D. Rajput, Munna Singh, Jaya Arora, Tatiana Minkina, Yang-Rui Li

**Affiliations:** 1 Key Laboratory of Sugarcane Biotechnology and Genetic Improvement (Guangxi), Ministry of Agriculture and Rural Affairs/Guangxi Key Laboratory of Sugarcane Genetic Improvement, Sugarcane Research Institute, Guangxi Academy of Agricultural Sciences, Nanning 530007, China; drvermakishan@gmail.com; 2 Department of Botany, Mohanlal Sukhadia University, Udaipur 313001, Rajasthan, India; abhijoshi2015@gmail.com (A.J.); jaya890@gmail.com (J.A.); 3 Institute of Biotechnology, Guangxi Academy of Agricultural Sciences, Nanning 530007, China; luckytian6688@163.com; 4 Academy of Biology and Biotechnology, Southern Federal University, 344090 Rostov-on-Don, Russia; rajput.vishnu@gmail.com (V.D.R.); tminkina@mail.ru (T.M.); 5 Department of Botany, University of Lucknow, Lucknow 226007, Uttar Pradesh, India; drmunnasingh@yahoo.com; 6 College of Agriculture, Guangxi University, Nanning 530004, China

**Keywords:** antioxidant enzymes, nano-fertilizers, photosynthetic capacity, plant nutrition, sustainable agriculture, stress resistance

## Abstract

Nano-fertilizers (NFs) significantly improve soil quality and plant growth performance and enhance crop production with quality fruits/grains. The management of macro-micronutrients is a big task globally, as it relies predominantly on synthetic chemical fertilizers which may not be environmentally friendly for human beings and may be expensive for farmers. NFs may enhance nutrient uptake and plant production by regulating the availability of fertilizers in the rhizosphere; extend stress resistance by improving nutritional capacity; and increase plant defense mechanisms. They may also substitute for synthetic fertilizers for sustainable agriculture, being found more suitable for stimulation of plant development. They are associated with mitigating environmental stresses and enhancing tolerance abilities under adverse atmospheric eco-variables. Recent trends in NFs explored relevant agri-technology to fill the gaps and assure long-term beneficial agriculture strategies to safeguard food security globally. Accordingly, nanoparticles are emerging as a cutting-edge agri-technology for agri-improvement in the near future. Interestingly, they do confer stress resistance capabilities to crop plants. The effective and appropriate mechanisms are revealed in this article to update researchers widely.

## 1. Introduction

Agriculture is the economic backbone of developing countries and also provides food for better living globally [1,2]. Climate change in ecosystems operates through biotic and abiotic stresses [3,4] which impair the delicate balance of environment linked with food production and may cause crop failure [2]. Our global food demand is anticipated to climb by approx. 70% in 2050 as a result of the increasing population [5,6]. It is necessary to ratify innovative and futuristic agri-technologies to achieve global food security with improved plant productivity [6], because environmental difficulties may arise primarily due to the inefficiency of various farming operations based on conventional fertilization practices [7]. The nutrient utilization efficiency (NUE) seems to be an important metric for assessing agricultural production and nutrient bioavailability in plants [8,9]. Fertilizers offer nutrients to optimize crop productivity, usually being applied through soil surface broadcasting or mixed with irrigation, which may cause damage to the atmosphere, water bodies, and the ecosystem [10,11,12,13,14]. Hydroponic techniques are used to grow some crops under greenhouse conditions, at the expense of about 10 times greater water and energy consumption, found to be neither economical nor sustainable [7,15,16]; the addition of fertilizers can cause eutrophication and ground water contamination, which may badly impact public health [17,18].

**Figure 1:**
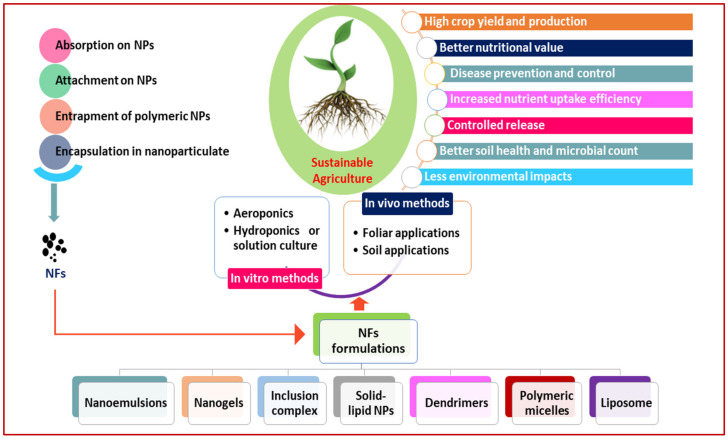
An overview of nano-fertilizer application in agriculture. NPs = nano-particles; NFs = nano-fertilizers.

**Figure 2:**
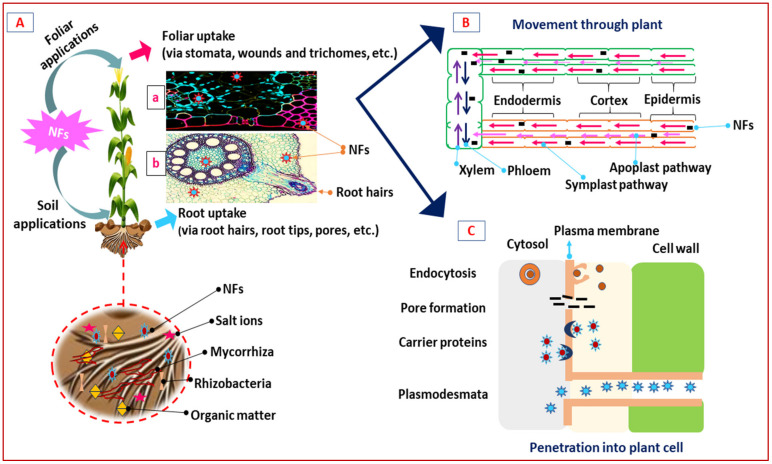
Uptake of NFs via various channels and their translocation paths across multiple plant sections are depicted schematically. (**A**) NF traits affect absorption and translocation in plants: (**a**) T.S. of maize leaf; (**b**) T.S. of maize roots (both images were taken from public databases and are freely accessible). (**B**) NFs may use apoplastic-symplastic pathways for moving up and down. (**C**) Various strategies were proposed for the internal distribution of NFs inside the cells through endocytosis and pore formation mediated by carrier proteins via plasmodesmata.

**Figure 3:**
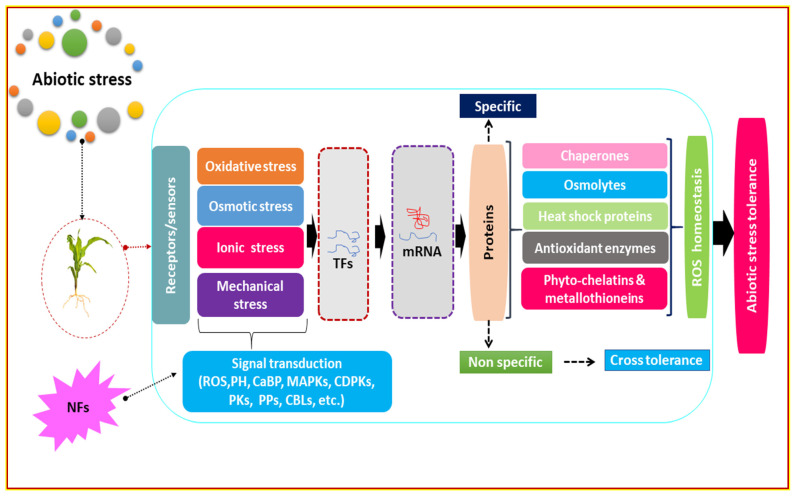
NFs with a variety of defense mechanisms in plants under stress. Early prevalence of stress sensing via receptors/sensors cascades the downstream stress response by ROS, CaBP (Ca^2+^ binding proteins), and plant hormones. Signal extension and transduction is carried out by secondary messengers, i.e., MAPKs (mitogen-activated protein kinases), PKs (ROS-modulated protein kinases), PPs (protein phosphatases), CDPKs (calcium-dependent protein kinases), etc. Signaling causes various regulation of transcription factors (TFs) and stress-responsive genes. Control of TFs and genes linked with physiological, biochemical, and molecular responses may adjust to fine-tune enhanced stress resistance capacity.

In such a scenario, newer agricultural interventions need to be implemented to fulfill the desire of our global food system [7] along with the safety of ecosystems, biodiversity, and the climate [12,13], as fertilizer use is proposed to be nearly 20% less by 2030 [19] through best farming practices to ensure approx. 85% of global agricultural production in the next 10 years [20,21]. Future agriculture may be based on the use of nano-enabled fertilizers in various ways, as nanotechnologies are becoming widely accepted in agriculture to enhance crop yields with a healthy agro-ecosystem under environmental adversities [22,23,24,25,26]. We examined recent trends in deploying engineered nanomaterials (ENMs) in agriculture, emphasizing nanotechnology-enabled fertilizers called “nano-fertilizers” and nano-pesticides with their improved delivery systems. The efficacies of the ENMs used are scrutinized, upgraded, and pooled herein (Figure 1, Figure 2 and Figure 3 and Table 1) to enrich researchers’ understanding.

## 2. Agriculture and Nano-Fertilizers

Nano-fertilizers are important in modern agriculture, having appropriate formulations and delivery mechanisms to ensure optimal uptake/usage in plants [2,12,27]. These nanoscale fertilizers reduce nutrient losses due to leaching, and chemical alterations can be avoided—to enhance nutrient use efficiency and environmental quality [14,28] by exploring NPs based on various metals and metal oxides for application in agriculture. The nanoscale particles are smaller in size and may be absorbed with different dynamics from those in bulk particles or ionic salts, which has significant benefits [29,30,31,32,33,34,35,36,37,38,39,40,41], as shown in Table 1. The usage of nano-enabled fertilizers may improve nutrient delivery efficiency in plants [37], as nano-fertilizers have demonstrated a boost in productivity by ensuring targeted delivery/gradual release of nutrients and reducing fertilizer application with an increase in NUE [42]. The reduced size of nano-fertilizers through physical/chemical means enhances their surface–mass ratio in order to allow an increase in absorption of nutrients by roots, as shown in Figure 1.

## 3. Nano-Fertilizers Mitigate Abiotic Stresses

ENMs have dramatically extended an increase in the functionality of biological systems due to their nanoscale size and vast surface area, which support plant growth and development [12,43,44,45] under biotic and abiotic stresses [13,46], viz., drought, salinity, alkalinity, temperature, minerals and metal toxicity [47]. Photosynthesis is a critical metabolic activity in plants and is found to be the most vulnerable to stress, viz., nutritional deprivation, salinity, drought, and heat, as photosystem II (PS II), rubisco, and ATP synthase become the primary targets [12]. Plant defense responses to abiotic stress have been established with SiO_2_ NPs to improve transpiration/ water-use efficiency (WUE), photosynthetic pigments, and carbonic anhydrase activities in pumpkin plants [13,48]. TiO_2_ has been discovered to alter photoreduction activity and block linolenic acid in the electron transport chain (ETC) located in chloroplasts for oxygen evolution [49]. Cell organelles produce reactive oxygen species (ROS) in stressful situations as the primary symptom of abiotic stress. Plants have the enzymatic machinery to deal with the oxidative stress they are subjected to by the environment. On the other hand, plants suffer the effects of such a situation when the defense system fails. By activating specific genes, collecting osmolytes, and supplying free nutrients and amino acids, NMs serve to alleviate stress, as shown in Figure 1, Figure 2 and Figure 3 and Table 1.

### 3.1. Uptake and Accumulation Mechanisms of Nano-Fertilizers from Soil to Plants

The dispersion, aggregation, stability, immobilization, bioavailability, and transport of NPs are influenced by the physicochemical properties of the soil, i.e., texture, structure, clay minerals, pH, cation exchange capacity, soil organic matter, and microbial population [50]. Due to the surface charge effect, dissolved organic matter impacts the aggregation, mobility, stability, and binding nature of NPs [51]. Nano-fertilizers may be applied to soil or foliage directly. The foliar entry comes first, followed by the root entry. In foliar application, provided by spraying the green canopies/leaves, uptake occurs mainly via the cuticle, stomata, and hydathodes, whereas root application acquires access via root tips, lateral roots, root hairs, and rhizodermis. NF foliar application is preferred during poor soil and weather conditions [2] (Figure 1 and Figure 2; Table 1). The flow of water and solutes through the soil is described by the Richards equation, and the convection–dispersion equation with different empirical models using the Michaelis–Menten equation [52,53].

Absorption increases in a nonlinear pattern as the nutrient concentration rises, approaching the highest uptake. The kinetic parameters of the Michaelis–Menten equation change depending on plant type, duration, soil heat, and related factors. The first nutrient transport model for plant tissues was the steady-state source–sink model with flow driven by an osmotically created pressure gradient [54,55] influenced by diffusive transport factors [56]; still, efforts are needed to acquire a perfect uptake model/mechanism for NPs in plants.

### 3.2. The Role of Nano-Fertilizers on Uptake of Water with Minerals

It is essential to assess the accumulation and translocation of NFs from the soil to plants. This information may help to explore the most appropriate ways and means for plants, as NFs/NPs prefer to enter via xylem uploading and may also be administered exogenously via phloem loading [57]. The uptake and distribution mechanism of nutrients through NFs in plants may be affected by the composition of NFs/NPs, their particle size, the dynamics of physiological processes, and pore diameter (5–20 nm) of the cell wall [57,58,59], while uptake, absorption, and trafficking of NPs inside plants occur with tremendous freedom (Table 1). Surface receptors, transporters, and membrane proteins were found to regulate their energy levels and surface charge [60,61]; in addition, regulated trafficking occurs through the cell wall and plasma membrane, as shown in Figure 2 and Figure 3 [62,63]. Plant cell walls are negatively charged and act as a surface for ion exchange, perhaps promoting the penetration of cationic rather than anionic NPs [64]. Thus, negatively charged NPs have higher transport efficiency, with improved rate of internalization and translocation [65]. In this scenario, positively charged CeO NPs strongly adsorbed onto the root surfaces (negatively charged), whereas negatively charged CeO_2_ NPs demonstrated limited root accumulation but increased shoot internalization, mostly by overcoming electrostatic resistance [66].

### 3.3. Impact of Nano-Fertilizer on Photosynthetic Leaf Gas Exchange Capacity

Upon use of nano-fertilizers, a considerable increase was achieved in the physiological and biochemical indices of crop plants. A biocompatible magnetic nano-fluid (MNF) enhanced the favorable effect on total chlorophyll content in sunflower leaves [67]. The foliar spray of nTiO_2_ enhanced photosynthetic pigments in *Zea mays* found to be associated with improved crop yield [68]. Jan Mohammadi et al. [69] found that the foliar application of nTiO_2_ barley boosted anthocyanin and photosynthetic pigments, rubisco activity, and photosynthetic efficiency. The use of nTiO_2_ in spinach boosted plant performance and enhanced nitrogen metabolism, protein levels, and green pigments up to 17-fold and the photosynthetic rate by approx. 29% [70,71]. The application of nano-Zn fertilizer decreased the peroxidase (POD), catalase (CAT), and oxidase enzyme activities in cotton and soybean crops but increased polyphenol content [72,73]. An increase in photosynthetic pigments, total soluble leaf protein, and dry mass of the plants was observed after foliar use of Zn NF in *Pennisetum glaucum* [74]. In savory plants, nano-Zn treatment-induced chlorophyll, essential oil, and P content [75] were observed, as an increase in the antioxidant capacity of rice [73]. Antioxidants are secondary metabolites produced by plants under adverse situations, i.e., drought, salt, and nutritional deficiency. The NFs supply enough nutrients to improve antioxidant activity in plant cells [76] due to an enhancement in photosynthetic responses generated by nTiO_2_ foliar use [77], with enhanced capacity of photo assimilation by leaves and grain yield [69]. The application of nTiO_2_ increased plant fresh and dry mass by enhancing photosynthetic capacity and nitrogen metabolism [74] through enhancing pigment formation and conversion of light energy into biochemical energy via improved photophosphorylation, which also upregulated biological carbon sequestration through the Calvin cycle in more than 95% of plants. The photocatalytic activity of nTiO_2_ in nanoform enhanced *Zea mays* biomass and productivity [68], as shown in Table 1. Si NPs increased photosynthetic efficiency by enhancements in the photosynthetic efficiency of PS II and in the performance index and photosynthetic pigments [78].

### 3.4. The Interactive Role of Nano-Fertilizers and Plant Growth—Biomass and Productivity

Morphological characteristics of nanocarriers may influence nutrient transport through the surface of the membranes, which is crucial in order to demonstrate the application and usefulness of nano-fertilizers, as shown in Table 1. It was found that nCHT has a good effect on morphological and physiological features in both germinating seed and foliar treatment to boost the growth of seedlings, biomass, germination capacity, and seed vigor index in chickpea, maize, and tomato seedlings [28,79,80]. Plant physiology and performance was found to be improved by increasing the density of certain nanoparticle surfaces [3]; application of nano-Zn resulted in significant modifications in rice biomass, relative water content [81], sunflower biomass [82], wheat grain yield under stress [83], and maize yield under drought [84] by increasing NUE [85] through alteration in physiological characteristics [86], including cell division, cell wall extension, and aquaporin in tobacco [87], shown in Table 1 and Figure 1, Figure 2 and Figure 3. NMs were also associated with developing seed vigor, as they may penetrate the seeds’ hard coating to allow water for the germination process. Thus, seed priming appears to be a promising procedure for high-yield value crops prior to sowing [88,89]. NPs, once delivered inside the cytosol, may interact with cellular machinery such as chloroplasts. Mesoporous silica nanoparticles (MSNs) enhance photosynthesis by acquiring adequate light-harvesting chlorophyll–protein complexes [13] and also do not cause stress in plants, indicating that they may be safe in such a type of smart delivery [90]. Silica is important for plant nutrition, as its deficiency makes plants weaker and more vulnerable to environmental stresses [12,91].

Depending upon their additive concentration and size, NPs might have a favorable or negative effect on plants by acquiring larger particles of TiO_2_ NPs [92]. These NPs may influence miRNA levels, triggering plant growth-promoting pathways [93]. At lower concentrations, Fe NPs had a favorable effect in *Capsicum annum*, promoting performance by increasing the number of chloroplasts and grana stacking to acquire stable and functional PS IIand ensure photo-bioenergetics [94]. Seed germination in *Vigna mungo* and *Hordeum sativum* distichum is affected by other metal oxides associated with ZnO and Cu NPs [95,96], and polyvinylpyrrolidone (PVP) protects *Pisum sativum* [97]. Chitosan, another important and biocompatible NP, influences germination percentage and morphology in wheat plants by increasing levels of IAA even at low concentrations [98]. The nano-nitrogen, phosphorus and potassium (nNPK) formulation may easily permeate the leaves through stomata, improving wheat gas exchange and leaf growth [99]. Jaberzadeh et al. [100] discovered that the foliar treatment of nFe (2%) boosted grain production. Similarly, manganese (Mn) NPs were used to boost output and yield components in *Vigna radiata* [101], followed by improved crop yield in peanut upon application of nFe, nMn, and nZn [102,103,104]. The foliar use of nano-chelated molybdenum (Mo) boosted morphological and physiological traits along with productivity in *Arachis hypogaea* [105,106], as shown in Table 1.

### 3.5. Influence of Nano-Fertilizer on the Regulation of Plant Hormones

Plant hormones are important in times of external stress and help plants to adapt under changing environmental conditions [107,108]. Thus, they play a crucial role in plant responses to unfavorable environmental conditions, viz., water deficit, shade, waterlogging, and cold temperature, mostly by slowing plant performance and refocusing its attention on surviving the stress response [109]. Phytohormones may also be delivered in *A. thaliana* with drought resistance ability [12,110]. The loss in auxins, cytokinins, and salicylic acid is caused by NPs, implying a hormonal imbalance in plants that affects general metabolism [111,112]. Zinc may stimulate important enzymes associated with biochemical processes, such as glucose and protein growth regulator metabolism, pollen production, and membrane integrity [113,114], and also terminal oxidase in mitochondria in order to face environmental adversities for survival, as nZn fertilizer may enhance plantgrowth-promoting hormones [46], as shown in Table 1.

### 3.6. Defense Mechanisms

When compared to Zn alone, ZnNPs enhanced Zn concentration and protein and carbohydrate metabolism but decreased the P content in wheat grains [115]. According to Burman et al. [116], the favorable response of nano-ZnO in chickpea may be related to low ROS (reactive oxygen species) levels for lowering lipid peroxidation and the activities of prominent antioxidative enzymes. In addition, Zn supplementation improves auxin production, promoting mineral absorption, cell division, and plant growth [114,117]. It also allows plants to retain the integrity of the plasma membrane [118] and the operation of the mitochondrial electron transport chain for liberating energy through oxidative phosphorylation linked with ATP synthase. Inadequate Zn may decrease IAA concentration in tomato plants [119]. Rezaei and Abbasi [72] found that applying nano-chelate Zn to cotton plants improves physiological processes by increasing chlorophyll content and antioxidant activities of CAT, POD, and polyphenol oxidase. The engineered TiO_2_ NPs could achieve improved photosynthesis as well as seed germination, plant development, and plant pest control in *Lettuce* [120,121,122,123,124], *Lemna minor* [125], *Solanum lycopersicum* [30], *Triticum aestivum* [126], *Citrullus lanatus* [127], *Phaseolus vulgaris* [128], and *Panicum miliaceum* [129,130], with increased nitrogen metabolism and ribulose-1,5-bisphosphate carboxylase/ oxygenase (rubisco)—encoded by the rbcL and rbcS genes located on cpGenome and nGenome and associated for contributing the LSU and SSU subunits of the proteins, biologically operating as enzyme biomolecules to ensure CO_2_ assimilation/Calvin cycle—perhaps the major cause of enhanced photosynthesis and plant productivity [12,49,70,71,124,131].

Plants create ROS mostly as a result of metabolic processes [132]. During metabolic processes, i.e., respiration and photosynthesis, plants regularly produce ROS in chloroplasts, mitochondria, peroxisomes, and other cell locations [132]. ROS are signaling molecules associated with plant development and defense at low levels and may also cause damage to cell membranes, DNA, protein, and other cell functions to impair plant growth [132,133] (Figure 1 and Figure 3; Table 1). Increasing the functional and structural protectants, such as suitable solutes (osmolytes) and antioxidative enzymes, may extend stress resistance capacity [134]. Antioxidative enzymes, i.e., SOD, CAT, APX, GR, GPX, and POD, and nonenzymatic low-molecular-weight metabolites primarily scavenge ROS in plants. Increased antioxidants may allow the formation of ascorbic acid and polyphenols, which may neutralize ROS to reduce oxidative stress. During stress, metabolic processes associated with ROS scavenging, i.e., shikimate-phenylpropanoid biosynthesis and ascorbate and aldonate metabolism, were found to be stimulated [135] to induce plants’ ability to scavenge ROS; by applying NMs with antioxidative enzymes, this may extend tolerance towards environmental stresses [133] with better plant performance and productivity (Figure 3).

### 3.7. Stimulation of Enzymatic and Non-Enzymatic Activities

NPs have some harmful effects on plants along with their beneficial aspects and may be used to bring health advantages to plants (Table 1). Because of their potential interaction at the nano–bio interface, these NPs have the potential to improve plant tolerance to various abiotic stresses [136] (Figure 3), as abiotic stress may cause generation of ROS, which lowers photosynthetic capacity and may lead to oxidation of biomolecules and peroxidation of biological membranes [137]. CeO NP mimics SOD activity and creates H_2_O_2_, but it also mimics CAT activity and demonstrates the scavenging action at a minimum ratio of Ce^3+^/Ce^4+^ [9,138,139,140]. Nano-TiO_2_ may upgrade plant hydration by boosting the nitrate reductase (NR) activity, leading to increased osmolyte accumulation. Increased NR enzyme activity leads to the generation of nitric oxide (NO), which triggers the synthesis of proline and glycine betaine [141]. In plants, TiO_2_ NPs tend to demonstrate enzymatic and non-enzymatic stress protection. TiO_2_ NPs may also regulate enzymes such as glutamate hydrogenase, glutamine synthase, and others, allowing the accumulation of additional nutrients and the generation of essential oils [142]. ROS generation seems to be the toxic mechanism of NPs, and various forms of NPs may produce various types of ROS by decreasing oxygen molecules. Reactive oxygen species are the byproducts of oxidative cellular metabolism, the bulk of which is carried outby mitochondria and which may give four types, the hydroxyl radical (OH^–^), the superoxide anion radical (O^2−^), hydrogen peroxide (H_2_O_2_), and singlet oxygen (_1_O^2^) [143,144]. The chemical composition of ENMs determines the quantity of ROS they produce [145]. ROS are produced due to NP accumulation and are responsible for cellular oxidative stress and the genesis of nanotoxicity, including DNA injury, manipulation of cell signaling, cell mortality, apoptosis, and cytotoxicity [146,147].

So far, studies have revealed that ROS regulate the physiology of cells and mechanisms by altering numerous signal transduction pathways in different cell types and systems [148]. Under normal circumstances, the generation of ROS in microbial cells was found to be balanced. This unbalanced environment causes oxidative stress, which destroys the different components of microbial cells. The redox balance of the cell favors oxidation with more ROS. It has been established that oxidative stress has a role in changing the permeability of cell membranes and generating microbial cell membrane injury [143,149]. ROS have also been proven to have a crucial interactive function between DNA and microbial cells, boosted oxidative protein gene expression being an important factor in microbial cell death. It may degrade proteins and impair the periplasmic enzymes necessary for microbial cells to balance their morphological and physiological functions [150], as shown in Figure 1, Figure 2 and Figure 3. One of the most effective ways to reduce the negative consequences of these pressures may be the use of NMs [151], because they may mimic enzymes such as POD, SOD, and CAT, which constantly scavenge ROS [81]. Si NPs stimulate the antioxidative system in stressed plants [12,152]. NPs may enhance enzymatic activities correlated to stress resistance, because they have more specific reactive surface areas. The loss in MDA accumulation in Si NP-applied plants was found to be significantly correlated to their membrane stability index [78] (Figure 3; Table 1).

### 3.8. Role of Nano-Fertilizers on the Expression of Stress-Responsive Genes

For DNA tagging and cleavage, interactions of NMs with nucleic acids have been employed. In contrast to the favorable applications of DNA–nanomaterial conjugation, fullerenes have been discovered to bind DNA and produce strand deformation, which may negatively influence molecule functionality and stability. Some NPs may indirectly damage DNA by generating ROS, resulting in cross-linking and DNA strand breakage [153]. The cells taking up nano-ions expressed continuous DNA alterations, identified during gene polymerization in vivo by the polymerase chain reaction (PCR) [154]. The oxygen radicals in TiO_2_ NPs, employed in sunscreen, may nick supercoiled DNA. Photosensitive fullerenes may cleave ds-DNA when exposed to light, with potential mutagenic functions [155]. Nanoparticles and their ions may interfere with DNA replication and expression of genes, as Ag ions are found to prevent DNA replication [153]. AgNPs bind to DNA in the cytoplasm of *E. coli*, impairing DNA replication [154]. In *Pseudomonas stutzeri*, *Azotobacter vinelandii*, and *Nitrosomonas europaea*, sublethal doses of Ag NPs caused no effect on N_2_-fixing or N_2_-denitrifying genes; nevertheless, other nitrification-related genes, such as amoA1 and amoC2, increased in *N. europaea* [156].

Microarray research of *E. coli* and Ag NPs reveals that NPs may greatly impact the bacterial transcriptome. Other molecular abnormalities are suggested by the stimulation of stress-related genes as well as genes for S, Cu, and Fe balance. The activation of nitrification genes without stimulation of denitrification genes (transformation of NO_3_ to N_2_) could affect the availability of N_2_ and the buildup of NO_3_ for later fixation. Ag NPs also alter other metal-regulated genes, suggesting that they have an impact on cellular metal homeostasis [157]. CuO NPs also reduced the output of luminous pyoverdine siderophores in *P. chlororaphis* by inhibiting the gene expression associated with their periplasmic maturation and secretion [158]. Ag NPs may alter other proteins related to metal detoxification, oxidative stress resistance, elongation and processes of transcription, cytoskeleton remodeling, protein loss, and cell division [159].

## 4. Long-Term Application of Nano-Fertilizers and Its Responses in Agriculture

Agriculture may experience environmental adversities in times to come, impairing our food security for a rapidly growing global population. Modifying current fertigation procedures [160], we may explore one of the possibilities for increasing plant performance, biomass, plant productivity, and eventually grain yield as well. The excessive use of chemical fertilizers may be harmful to the health of mankind, animals, the health of plants/crops, and the environment. The inclusion of nano-fertilizers could be a promising, fruitful solution to these issues. The use of NFs was found to be one of the most effective methods for increasing resource efficiency, plant production, and lowering pollution levels [161]. Thus, NFs may replace the application of regular fertilizers by providing a suitable approach to improving the agricultural products [162], as shown in Table 1.

**Table 1:** Impact of nanoparticles/nano-fertilizers on crops under unfavorable environmental variables.

NPs	Plant	Application Type	Concentration Range	Impacts	Source
nCeO_2_	Barley (*Hordeum vulgare* L.)	Soil	0–500 mg kg^−1^ soil	Improved plant performance, enhanced Ce accumulation in grains, and P, K, Ca, Mg, S, Cu, Fe, Zn, Mn, Al, amino acids, fatty acids, methionine, aspartic acid, threonine, tyrosine, arginine, and linolenic acid.	[163]
	Wheat (*Triticum aestivum* L.)	Soil	0–500 mg kg^−1^ soil	Enhanced overall plant fitness and productivity as compared to normal plants—increased Ce uptake in roots but no change in leaves, hull, and seeds.	[164]
	Wheat (*Triticum aestivum* L.)	Soil	0–400 mg kg^−1^ soil	Reduced photosynthetic pigments and seed protein, antioxidant enzyme activities upregulated. No significant effects on plant biomass and productivity.	[165]
	Cucumber (*Cucumis sativus* L.)	Soil	400 mg kg^−1^ soil	No change in starch level but changed carbohydrate pattern. Enhanced globulin and reduced glutelin content.	[166]
	Cilantro (*Coriandrum sativum* L.)	Soil	0–500 mg kg^−1^ soil	Higher content was found in Ce, CAT in the stem, and APx in roots.	[167]
nCuO	Tomato (*Solanum lycopersicum* L.)	Foliar	50–500 ppm (particle size 50 nm)	Enhanced vitamin C, lycopene, ABTS, CAT, and SOD and reduced the APX and GPX activities. Increased Cu accumulation in tomato fruits.	[168]
	Tomato (*Solanum lycopersicum* L.)	Soil	0.02–10 ppm	Improved plant growth, development, productivity, and fruit quality. Enhanced the lycopene and antioxidant capacity.	[169]
	Cucumber (*Cucumis sativus* L.)	Hydroponic	10–20 ppm	Increased ROS, phenolic components, amino acids, antioxidant enzymatic systems, and decreased citric acid level.	[170]
	Cucumber (*Cucumis sativus* L.)	Soil	40 nm (particle size)	Fruit metabolites were changed as compared to control plants. Sugars and organic, amino, and fatty acids were enhanced.	[171]
	Tomato (*Solanum lycopersicum* Mill.)	Soil	10–100 mM	Enhanced plant biomass and growth characteristics. Upregulated photosynthetic pigments, leaf gas exchange responses, and enzymatic activities.	[172]
nCuO, nAl_2_O_3_, nTiO_2_	Onion (*Allium cepa* L.)	Petriplate	0–2000 µg mL^−1^	Significantly affected the mitotic index. ROS activities enhanced in onion roots. Enzymatic activities increased, i.e., CAT and SOD in all applied NPs.	[173]
nCu/ kinetin	Kidney bean (*Phaseolus vulgaris* L.)	Soil	50, 100 mg kg^−1^ soil	The chlorophyll content and nutrient elements, Ca, Mn, and P, were reduced and root Cu accumulation enhanced.	[174]
nCu–chitosan	Tomato (*Solanum lycopersicum* L.)	Soil	0.3–0.015 M	Increased plant performance, productivity, stomatal conductance, and leaf CAT and fruit lycopene level.	[175]
nCu, nFe, nCo (Metal NPs)	Maize (*Zea mays* L.)	Soil irrigation	3–5 ppm	Positively enhanced the seed germination frequency, time, and early growth, enzymatic activities, and metabolism of SOD in plant leaves to stress resistance capacity.	[176]
nSiO_2_	Maize (*Zea mays* L.)	Hydroponic	20–40 nm	Enhanced germination (%) rate, biomass, Si uptake, and nutrient uptake	[177]
	Soybean (*Glycine max* L.)	Soil	30–50 nm (particle size)	Reduced the toxic effects on plant performance and reduced Hg uptake in the epidermis and pericycle of the plant roots and leaves. Increase leaf gas exchange and enzymatic responses.	[178]
	Peregrina (*Jatropha integerrima*)	Foliar	1–2 mM	Increased growth characteristics, biochemical profile, meanwhile reduced uptake of Na, Cl, total phenolics, and flavonoid contents in the plant leaves.	[179]
	Tomato (*Solanum lycopersicum* L.)	Petriplate	0.05–2.5 ppm	The germination rate, root morphology, and biomass were significantly enhanced after NPs. Gene expression was upregulated, i.e., in AREB, TAS14, NCED3, CRK1, and RBOH1, APX2, MAPK2, ERF5, MAPK3, and DDF2 decreased. The genes are significantly associated to nSi in plant’s response to enhance stress resistance capacity.	[180]
	Mahaleb (*Prunus mahaleb* L.)	Soil irrigation	10–100 ppm	Improved photosynthetic performance less impacted by stress when plants were pretreated with NPs at maximum treatment concentrations and upgraded nutritional level, i.e.,N, P, and K content.	[181]
	Faba bean (*Vicia faba* L.)	Soil	1–3 mM	Improved seed germination rate and duration, plant length, leaf RWC biomass, seed quality, and productivity and nutritional element status, i.e., N, P, K, Ca, and Na.	[182]
	Cucumber (*Cucumis sativus* L.)	Foliar	15–120 ppm	An enhancement in plant length, leaf number, areaexpansion, biomass, fruit weights, and quality as relative to control plants.	[183]
	Strawberry (*Fragaria × ananassa*)	Foliar and soil irrigation	20–80 ppm	Significantly enhanced the nutritional content, such as K, Ca, Mg, Fe, Mn, and Si, in plant stem but no changes in Zn and Cu content.	[184]
	Sugarcane (*Saccharum officinarum* L.)	Foliar	300 ppm	Enhanced photosynthetic efficiency, Fv/Fm variables, chlorophyll content, and PS II apparatus during cold stress conditions.	[185]
	Barley (*Hordeum vulgare* L.)	Soil	12–250 ppm	Significantly enhanced plant growth performance, chlorophyll content, leaf gas exchange, osmolytes, antioxidative enzyme activities, cell membrane efficiency, and profile of metabolites.	[186]
	Wheat (*Triticum aestivum* L.)	Hydroponic	10 µM	Alleviates harmful effects of UV radiation on plants.	[187]
	Marigold (*Tagetes erecta* L.)	Soil and foliar	100–600 ppm	Enhanced biometrics, physiological, biochemical, and flower traits, i.e., fresh and dry mass of flower, flowering duration, and days taken to first bud initiation, etc.	[188]
Biogenic amorphous silica (bASi)	-	Soil	1–15%	Increases soil water holding capacity (SWHC). Soil management can be modified to increase bASI level, increasing available water content in soils, and to reduce water stress capacity for plant growth and development.	[189]
nFe_2_O_3_	Soybean (*Glycine max* L.)	Foliar	0.25–1 M	Enhanced leaf biomass with seed weight in comparison to normal plants.	[190]
	Peanut (*Arachis hypogaea* L.)	Soil	2–1000 ppm	Improved plant growth characteristics, root morphology, and productivity. Enhanced photosynthetic pigments, Chl index, plant hormones, enzymatic activities, and Fe uptake.	[191]
	Tomato (*Solanum lycopersicum* L.)	Hydroponic	50–800 ppm	Improved germination of seeds, morphological traits, dry weight, and Fe uptake as compared to normal plants	[192]
nFeS	Mustard (*Brassica juncea* L.)	Foliar	2–10 ppm	Enhanced agronomic traits, photosynthetic pigments, membrane injury, nutrient assimilation, MDA, proline, and enzymatic activities versuswithout NP application. Activation of genes, i.e., rubiscosmall subunit (rubisco S), rubiscolarge subunit (rubisco L), glutamine synthetase (gs), and glutamate synthase (gogat).	[45]
nTiO_2_	Cucumber (*Cucumis sativus* L.)	Soil	0–750 mg kg^−1^ soil	Enhanced leaf greenness, CAT, and APx activity were reduced. Applied TiO_2_ increased Kand Plevels.	[193]
	Barley (*Hordeum vulgare* L.)	Soil	500–1000 mg kg^−1^ soil	Applied NPs found tostimulate plant performance by enhancing germination (%) as compared to normal and treated plants.	[39]
	Rice (*Oryza sativa* L.)	Soil	0–750 mg kg^−1^ soil	Enhanced plant performance, P level in roots to grains. Upregulated the level of metabolites, i.e., amino acids, palmitic acids, and glycerol level in rice seeds.	[194]
	Tomato (*Solanum lycopersicum* L.)	Soil	0–1000 mg kg^−1^ soil	Improved plant development uptake and accumulation of minerals.	[195]
	Tomato (*Solanum lycopersicum*L.)	Hydroponic	0.5–4 M	nTiO_2_ improved plant growth and development (approx. 50%) and significantly enhanced the leaf gas exchange, i.e., quantum yield, performance index, photosynthetic pigments, and expression ofPSIgene compared to normal plant growth conditions. Enhanced expressions of glutathione synthase and glutathione*S*-transferase in roots and leaves. Antioxidant activities increased in a dose-dependent method. Nutritional element significantly affected (P, S, Mg, and Fe content).	[196]
	Spinach (*Spinacia oleracea* L.)	-	0.25%	Enhanced electron transport rate (ETR) and the oxygen-evolving rate (OER) of PS II, enzymatic responses, reduced ROS level.	[197]
	Tomato (*Solanum lycopersicum* L.)	Foliar	0.05–0.2 M	Increased photosynthetic performance by regulating PS II energy dissipation and slightly reduced the *Fv/Fm* and electron transport rate in plant leaves.	[198]
	Wheat (*Triticum vulgare* L.)	Hydroponic	5–40 ppm	No significant effects on plant performance. Leaf photosynthetic pigments were reduced with increasing NP levels. Increased nutrient uptake and accumulation except for K level.	[199]
nTiO_2_-Activated carbon composite	Tomato (*Solanum lycopersicum* L.) and mungbean (*Vigna radiates* L.)	Foliar	0–500 ppm	Appropriate NP concentrations can enhance the rate of seed germination and minimize the germination period in tomato and mungbean.	[200]
nFe_3_O_4_	Cucumber (*Cucumis sativus* L.)	Hydroponic	50–2000 ppm	Improved plant growth, development, yield, and enzymatic responses, i.e., SOD and POD. Applied NPs enhance/balance the proper nutrient management to overcome food security and safety.	[201]
	Barley (*Hordeum vulgare* L.)	Hydroponic	125–1000 ppm	Increase plant growth, biomass traits, photosynthetic pigments, total soluble protein, and chloroplasts frequency. No toxic effects were found during the excess dose of NPs. Excess NP application reduced the CAT and H_2_O_2_ activities, and alteration was found in the photosynthetic genes of plant leaves.	[202]
nFe	Chili (*Capsicum annuum* L.)	Foliar	0.002–2 mM L^−1^	Low dose of nFe was noted to play positive role in plant growth and development. Enhanced chloroplast functional capacity and grana stacking. High dose of FeNPs found to have harmful effects on plants and can potentially stop the distribution of Fe nutrient.	[94]
nAg	Tomato (*Solanum lycopersicum* L.)	Seed	0.05–2.5 ppm	Enhanced the rate of germination (%), root morphology, and plant output. The expression of genes was found to be upregulated (AREB, MAPK2, P5CS, and CRK1), and few genes were noted as downregulated (TAS14, DDF2, and ZFHD1).	[203]
	Tomato (*Solanum lycopersicum* Mill.)	Soil irrigation	10–40 ppm	Applied NPs enhanced the fruit characteristics and plant performance.	[204]
	Soybean (*Glycine max* (L.) Mell.)	Soil	31.2–62.5 mg kg^−1^ soil	Negatively affected plant development and fixation of N.	[205]
nZnO	Maize (*Zea mays* L.)	Foliar	150–300 ppm	Enhanced maximum growth characteristics, physiological and biochemical activities during high pH treatment.	[206]
	Mungbean (*Vigna radiate* L.)	Petriplate	10–100 ppm	Enhanced germination rate, growth development, and nutritional elements.	[207]
	Tomato (*Solanum lycopersicum* Mill.)	Tissue culture	15–30 ppm	ZnO NPs alleviated the adverse effects of plants. Lower dose was more appropriate than the higher. Various cultivars found different tolerance capacity for stress.	[208]
	Maize (*Zea mays* L.)	Foliar	50–2000 ppm	Enhanced seed germination rate, seedling vigor index, biomass, productivity, and accumulation of Zn in grains.	[32]
	Peanut (*Arachis hypogaea* L.)	Soil irrigation	0–1000 ppm	Increased vegetation growth rate, morphological traits, photosynthetic content, crop productivity, and overall plant performance.	[209]
	Sweet basil (*Ocimum basilicum*L.)	Foliar	1000 ppm	Improved vegetative growth, development, essential oil productivity, biomass, and accumulation of Zn content.	[210]
	Peanut (*Arachis hypogaea* L.)	Soil	100–500 ppm	Morphological, yield, and biochemical traits, such as plant length, biomass, and pod numbers/weight. Photosynthetic pigments, total phenols, reducing and total soluble sugar were positively affected by the NP treatment.	[211]
	Sorghum (*Sorghum bicolor* L.)	Soil and foliar	6 mg kg^−1^ soil	Enhanced plant performance and yield component, uptake of N and K elements, improved grain nutrient profile and NUE as compared to normal plants.	[212]
nZn–chitosan	Wheat (*Triticum durum*)	Soil and foliar	20 mg g^−1^ soil (w/w)	Increased Zn accumulation in the plants cultivated under Zn-deficient arable land.	[213]
nChitosan	Barley (*Hordeum vulgare*L.)	Soil and foliar	10–100 ppm	Significantly enhanced the leaf areaexpansion, leaf greenness (Chl index), number of seeds/spikes, productivity, and harvest index relative to normal plants. nChitosan enhanced the LRWC, grain weight, grain protein, proline, and CAT and SOD activity.	[214]
nChitosan-NPK	Wheat (*Triticum aestivum* L.)	Foliar	500, 60, and 400 ppm (N, P, and K), 10, 25, and 100%	Enhanced growth, yield, and nutritional status as compared to normal plants.	[215]
nChitosan	Barley (*Hordeum vulgare* L.)	Soil and foliar	30–90 ppm	Positively enhanced the growth parameters, leaf chlorophyll index, RWC, yield, and biochemical activities.	[214]
nZ (Zein NPs)	Sugarcane (*Saccharum* spp.)	Hydroponic	0.88–1.75 mg mL^−1^	Uptake of significant amount of ZNPs in cane roots and the presence of Z particles in the epidermis and endodermis in the roots system of the sugarcane plant. Increased nutrient uptake in the plant system.	[216]
nAu	Thale cress (*Arabidopsis thaliana* L.)	Foliar	10–80 µg mL^−1^	Increased seed germination (%), growth, free radical scavenging responses. Potential approach to increase the seed productivity of plants.	[217]
	Brown mustard (*Brassica juncea* L.)	Foliar	0–100 ppm	Significantly enhanced the growth, biomass parameters, and total sugar level. Leafarea expansion was increased, but the mean area not affected.	[218]
Mn_3_O_4_	Cucumber (*Cucumis sativus* L.)	Foliar	1–5 mg plant^−1^	Significantly enhanced plant development, chlorophyll content, photosynthetic responses, and plant biomass. Increased endogenous antioxidative defense mechanisms.	[219]
nUrea modified with hydroxyapatite	Almond (*Prunus dulcis* L.)	Soil irrigation	25–100%	Applied NPs enhanced seed germination rate, plant height, perimeter, elongation of secondary and primary roots/plant, and the number of secondary roots, increasing seed moisture status.	[220]

Ironoxide nanoparticles may have a long history of use in a variety of fields, including catalysis and medicine [221]. Iron NPs applied to plants by irrigation of soil or spraying are absorbed and accumulated in *Zea mays* [222], *Cucurbita pepo* [223], and *Citrullus lanatus* [127,224]. Fundamental demonstrations of the effects of FeO NPs on a variety of plants, such as *Lactuca sativa* [121], *Triticum aestivum* [101], *Trifolium repens* [225], *Glycine max* [101,226], *Oryza sativa* [227], and *Arachis hypogaea* [191], reflected an idea that Fe NPs may improve a variety of morphological, physiological, biochemical, and yield attributes, as shown in Table 1. Plants may absorb FeO NPs as intact particles, and they eventually dissolve with positive effect on development. Thus, Fe NPs could be a good addition as a standard chelator for iron fertilizer. However, several aspects, such assoil properties, soil biogeochemistry, plant cultivars, plant growth stage/duration, NP exposure level, and physicochemical features, may limit these results, necessitating further validation with a variety of crops and soils, including subsequent effects on the food chain [12,46]. Notably, silica NPs also stimulate antioxidant enzymes to support improved seedling growth and tolerance under biotic and abiotic stresses [12,13,78,178,228]. They also inhibit Na uptake and its distribution while enhancing K uptake and accumulation [229,230]. As a result, the favorable effects of Si and Si NPs on plant growth and development may confer an ability to mitigate adverse impacts of climate change in years to come, for example, inconsistent rainfall, elevated or cold temperatures, and excess evaporation from the soil surface [78]. Si NPs may interact with plants directly or indirectly, causing morphological and physiological changes for upregulating stress resistance, which may sustain plant growth, development, performance, suitable canopy architecture, and rhizosphere and biosphere physiological acts assisting stress acclimatization [13,231], as shown in Table 1.

The potential of Si to influence the availability of P and boost the NUE, expressed as biomass produced per unit of a specific essential nutrient accumulated concerning N, has been shown [232,233]. As a result, Si supplementation in agricultural soils may eliminate the requirement of P and N fertilizer for crop plant production [234]. The wheat plants rapidly absorbed silicon, which was supplied to the substrate in the form of ENMs made up of amorphous pyrogenic hydrophilic SiO_2_ [233]. The maximum concentration of Si was found in vegetative tissue, i.e., leaf blades > leaf sheaths > culm and the lowest in grain followed by roots, increasing stomata density in the tissues. Wheat plants were shown to transfer around 90% of absorbed Si to the shoots, while root content remained low [233,235]. The addition of Si greatly boosts Pmobility by mobilizing Fe(II)-P phases from mineral surfaces. In phosphorus-deficient soils, it also stimulates soil respiration. It isa major factor for mobilizing phosphorus in Arctic soils, suggesting that this may also be important for sustainable management of phosphorus availability in soils [233,236].

Environmental protection, financial stability, and biological sustainability are more notable consequences of nano-goods supporting plant crops (Table 1). According to Tiwari et al. [237], NMs may boost plant stress tolerance, whereas nano-fertilizers may improve overall plant health. The specific biosensors may be associated with NFs to manage the delivery and bioavailability of nutrients based on plant types, growth stage, and agro-climatic zones. Above all, huge industrial set-up and vast transport using large quantities of energy may also be compressed in the event of global acceptance of NFs, as these are required in modest quantities compared to synthetic fertilizers for crop production [160,238].

## 5. Conclusions and Future Perspective

Plenty of ENMs have extended genuine promise in agriculture and plant/crop productivity. However, much more seems to be unestablished to reinforce scientific knowledge in order to produce another green revolution in years to come for the welfare of global food security under the era of climate change, associated with adverse environmental variables and increasing population in developing countries. As agricultural produce is essential to support and sustain life on planet Earth, recent advances of NFs may be explored to achieve precision agriculture with ecological and economical viability. It must be noted that the worldwide green revolution resulted in enhanced production of food grains at the cost of disproportionate use of artificial/synthetic fertilizers and pesticides, which severely impaired our ecosystem. Thus, both of these troubles associated with agriculture may be substituted in an eco-friendly way by using NFs and NPs to safeguard our ecosystem/agro-climatic zones. The fertilizers obtained from biological resources may have numerous advantages over synthetic fertilizers in maximizing crop expansion with nutrient utilization efficiency and mitigation of climate change. Interestingly, NFs have shown considerable promise as an innovative approach and may confer in vivo enhanced agri-potential by maintaining the physiological fitness of crop plants holistically to maximize their metabolic processes, viz., WUE, NUE, LUE, and CUE, integrated with plant performance and plant productivity/carbon economy, found to be essential for the exploration of another green/grain revolution to feed the population globally with a healthy ecosystem under climate change in the future.
